# A Case of Thrombotic Microangiopathy and Acute Sarcoidosis

**DOI:** 10.1016/j.chest.2022.06.023

**Published:** 2022-11-04

**Authors:** Anthony W. Martinelli, William Dunn, Mark E. McClure, Ieuan Walker, Andrew Stewart, Sumit Karia, Stephen D. Preston, Sathia Thiru, Nicholas Torpey, Sanjay Ojha, Emily Symington, James A. Nathan

**Affiliations:** a Department of Respiratory Medicine, Addenbrooke’s Hospital, Hills Road, Cambridge, England; b Cambridge Institute of Therapeutic Immunology and Infectious Disease, Department of Medicine, University of Cambridge, Cambridge, England; c Department of Haematology, Addenbrooke’s Hospital, Hills Road, Cambridge, England; d Department of Renal Medicine, Addenbrooke’s Hospital, Hills Road, Cambridge, England; e Department of Radiology, Addenbrooke’s Hospital, Hills Road, Cambridge, England; f Department of Histopathology, Royal Papworth Hospital, Cambridge, England; g Department of Histopathology, Addenbrooke’s Hospital, Hills Road, Cambridge, England

**Keywords:** Hemolytic anemia, ILD, MAHA, sarcoidosis, thrombotic microangiopathy, TMA, TMA, acute thrombotic microangiopathy

## Abstract

Although sarcoidosis is an established cause of multiorgan dysfunction, acute presentation with thrombotic microangiopathy resulting in severe renal and hematological sequelae has not been reported. We describe the case of a patient presenting with hypercalcemia, pancreatitis, and acute renal failure, followed by microangiopathic hemolytic anemia. Although there were no significant respiratory symptoms, thoracic radiology and mediastinal lymph node biopsy results were in keeping with sarcoidosis as the underlying cause of this multisystem presentation. Corticosteroids were commenced with clinical and biochemical improvement. This novel case highlights the need to consider sarcoidosis as part of the differential diagnosis for unusual multiorgan presentations and for early multidisciplinary involvement in such cases to permit optimal treatment.

Although pulmonary, cardiac, and neurological presentations of sarcoidosis are encountered frequently in clinical practice, renal and hematological sequelae of the disease are less well described. Sarcoidosis-associated kidney dysfunction in particular is usually attributable to granulomatous nephritis (renal sarcoidosis) or as a direct consequence of hypercalcemia (including nephrolithiasis and nephrocalcinosis).^1^^,^^2^ Hematological abnormalities—such as anemia and thrombocytopenia—are common in patients with sarcoidosis, but often mild in severity, incidental, and transient, whereas hemolytic anemia is rare and has only been described in a small number of case reports.^2^ The potential for sarcoidosis to manifest with a wide variety of symptoms, often seemingly unrelated, is also widely noted, but it remains of critical importance to confirm the diagnosis rapidly to institute treatment and prevent morbidity and mortality.^3^

## Case Report

**Figure 1:**
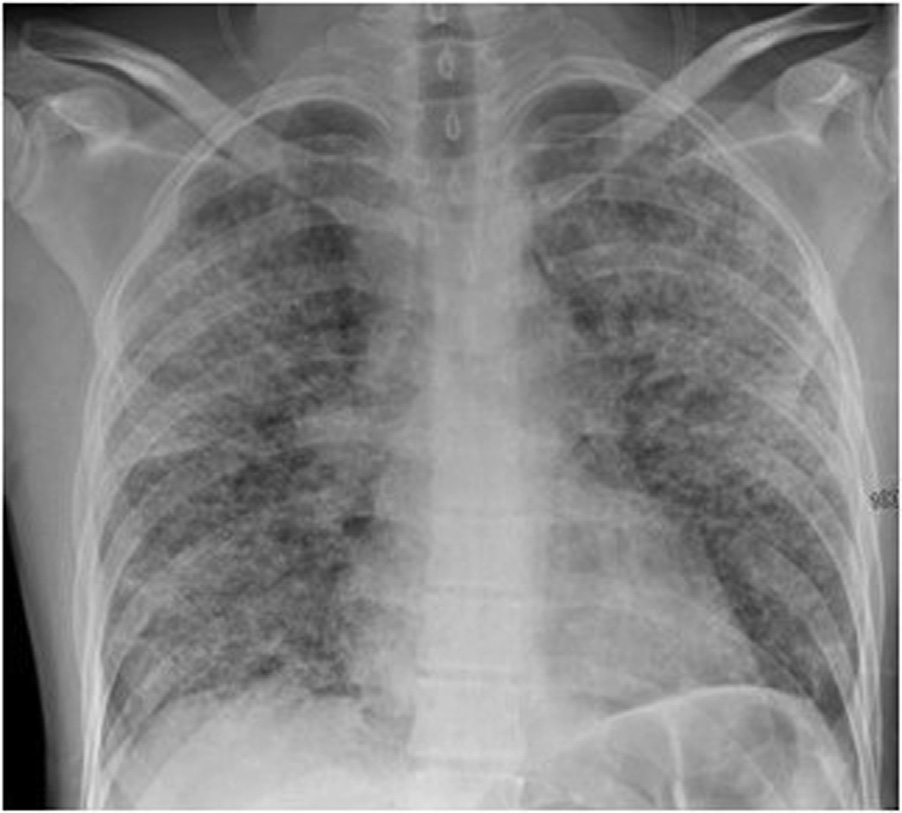
Chest radiograph on presentation to hospital without respiratory symptoms*.*

A 31-year-old man presented to the ED with epigastric pain and nausea, associated with generalized malaise and fatigue. The patient also described 2 days of mild, and 1 night of profuse, nonbloody diarrhea approximately 3 weeks before their attendance. He was a current smoker, working as a delivery driver, with no significant medical history and no regular prescribed medications. Over-the-counter medications included a multivitamin supplement and loperamide taken after the episode of profuse diarrhea. He had no history of illicit drug use. Initial investigations indicated acute kidney injury (Cr 241 μM) with hemoproteinuria, a raised amylase (191 U/L), and widespread pulmonary infiltrates visible on initial chest radiograph (Fig 1). The radiological changes were noted to be disproportionate to the minimal degree of subjective dyspnea and lack of any oxygen requirement (oxygen saturation 92% on air). IV fluids and antiemetics were commenced.

**Figure 2:**
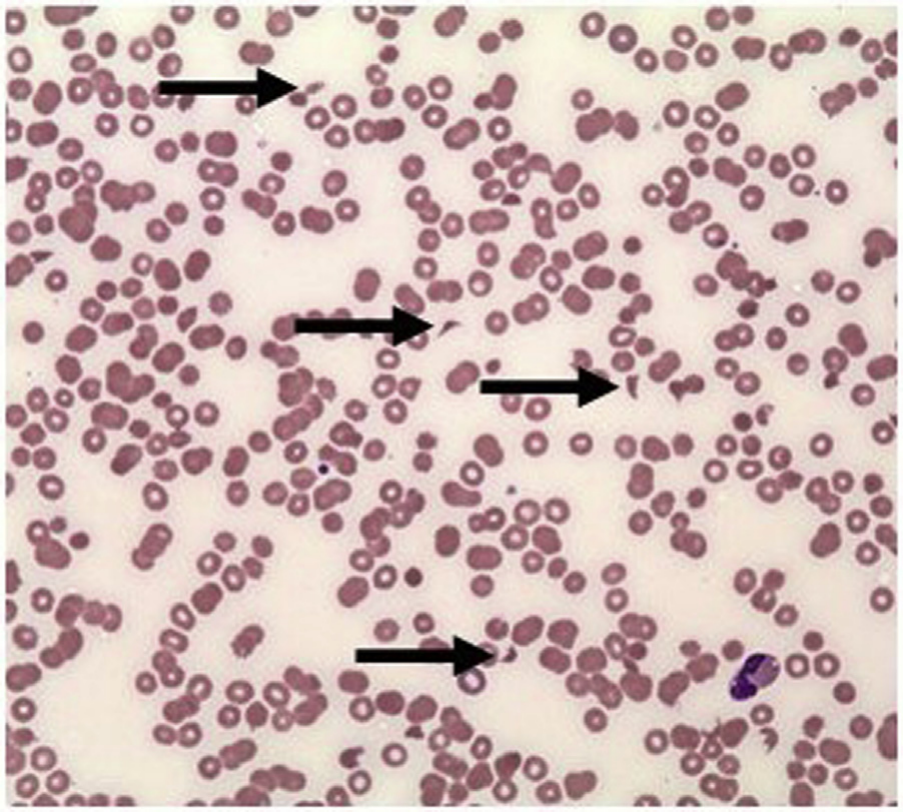
Blood film showing microangiopathic hemolytic anemia, with arrows noting schistocytes*.*

Two days after admission, the patient developed anemia (105 g/L) and thrombocytopenia (32,000 cells/μL) alongside an inflammatory response (c-reactive protein > 100 mg/L). A bone profile test revealed hypercalcemia (3.19 mM, corrected). In this context, a blood film was performed, which showed red cell fragmentation (more than four per high-powered field), consistent with a microangiopathic hemolytic anemia (Fig 2). The patient’s activated partial thromboplastin clotting time was normal (27.2 s), with prothrombin time only mildly prolonged (13.9 s) and a normal fibrinogen (1.61 g/L), excluding disseminated intravascular coagulation. The ADAMTS13 level was normal (58.0 IU/dL), excluding thrombotic thrombocytopenic purpura. The patient was transferred to a tertiary center, where zoledronic acid was administered for hypercalcemia.

**Figure 3:**
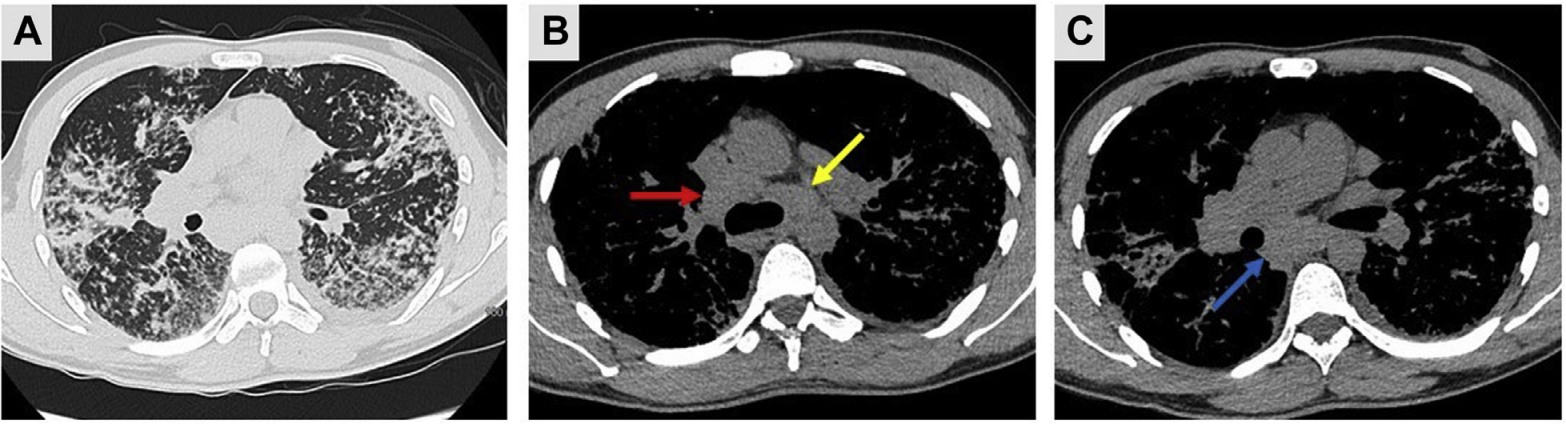
Cross-sectional imaging. A, CT scan of thorax (lung window) showing lung parenchymal changes. B, C, Non-contrast–enhanced CT scan of thorax (mediastinal soft tissue window) showing multiple enlarged (up to 2.8 cm) noncalcified thoracic lymph nodes involving stations 4R (red arrow), 4L (yellow arrow), and 7 (blue arrow).

Given the diagnostic uncertainty and multiorgan involvement, cross-sectional imaging was undertaken, showing marked perilymphatic ground-glass changes and nodularity in the midzones of both lungs, mediastinal lymphadenopathy, and splenomegaly (Fig 3). Investigations for immunodeficiency and autoimmune disease were negative, including antiphospholipid antibodies, antinuclear antibody, anti-neutrophil cytoplasmic antibody, anti-glomerular basement membrane antibody, and rheumatoid factor. No infective causes were identified. A bone marrow trephine showed trilineage hematopoiesis with normal megakaryocyte number and morphology and no evidence of an underlying bone marrow failure syndrome or hematological malignancy. The patient’s serum angiotensin-converting enzyme level was elevated (84 U/L; normal range, 0-52 U/L).

**Figure 4:**
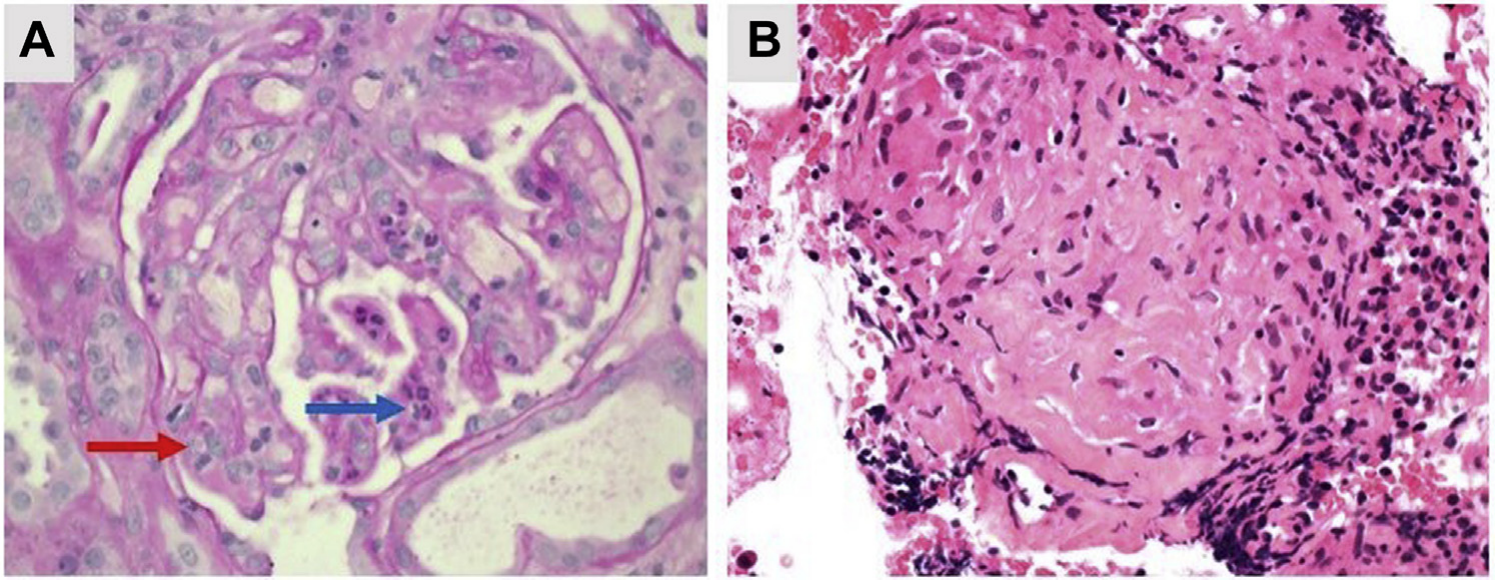
Histopathology results confirming diagnoses. A, Renal biopsy showing glomerular acute thrombotic microangiopathy with mesangiolysis: red arrow indicating capillary lumen occluded by endothelial cell swelling and the blue arrow indicating acute inflammatory cells in capillary lumen. B, Endobronchial ultrasound-guided transbronchial fine needle aspiration section showing a non-necrotizing granuloma*.*

Transfusion of packed RBCs and platelets allowed a percutaneous kidney biopsy to be performed, which showed severe acute thrombotic microangiopathy (TMA), with no evidence of granulomata or interstitial nephritis (Fig 4A). Given this biopsy result, alongside a peak serum creatinine of 304 μM, the treating team considered initiation of plasma exchange or prescription of the complement-inhibitor eculizumab for a presumed diagnosis of atypical hemolytic uremic syndrome. However, the patient’s renal function began to improve in the days after the biopsy, without requiring either of these interventions. Genetic testing later confirmed that the patient did not possess any gene mutations associated with atypical hemolytic uremic syndrome. A diagnosis of Shiga toxin–associated hemolytic uremic syndrome was also considered in light of the diarrheal prodrome, but a direct polymerase chain reaction assay for Shiga toxin from a rectal swab was negative.

With the cause of his pulmonary changes and hypercalcemia unconfirmed, the patient proceeded to urgent endobronchial ultrasound-guided biopsy of his enlarged mediastinal lymph nodes (stations 4R, 11R, 7R) and BAL of his middle lobe. The BAL, performed after the endobronchial ultrasound-guided biopsy, was blood-stained without prominent hemosiderin (indicating post-biopsy acute hemorrhage). The differential cell count was, as a result, difficult to interpret (lymphocytes 18%, macrophages 54%, neutrophils 25%, eosinophils 3%; based on a count of 300 cells). BAL microscopy and culture was negative, including for *Mycobacteria*. Critically, the lymph node biopsies identified non-necrotizing granulomata consistent with sarcoidosis (Fig 4B) and, as a result, the patient was commenced on oral prednisolone 40 mg/day in line with European Respiratory Society guidelines for treatment of this condition.^3^ On subsequent outpatient follow-up, the patient remained well. There was normalization of serum calcium, renal function, hemoglobin and platelet counts, along with improvement of his chest radiology, and pulmonary function tests showing a mild restrictive defect with loss of units in gas transfer (transfer capacity of the lung, 63%; carbon monoxide transfer coefficient, 81%).

## Discussion

To establish a final diagnosis for this case, we must consider whether the patient’s confirmed significant findings of granulomatous inflammation and TMA could be attributed to any other underlying condition. Both TMA and sarcoidosis can be associated with infection, but although hemolytic uremic syndrome is usually secondary to *Escherichia coli* or *Streptococcus pneumoniae*, sarcoidosis has more frequently been linked to previous infection with *Mycobacteria* and *Propionibacteria*.^4^^,^^5^ Extensive screens for an infective agent were negative. In particular, the negative rectal swab for Shiga toxin argued against an infective cause, although we acknowledge that the sensitivity of this investigation was limited by the need to take a rectal swab (rather than stool sample) because the diarrhea had completely resolved. Similarly, although both sarcoidosis and TMA can be associated with autoimmune disease, our tests for relevant autoantibodies were uniformly negative. Furthermore, the autoimmune diseases most commonly associated with sarcoidosis (autoimmune thyroid disease, Sjögren’s syndrome, and ankylosing spondylitis) are distinct to those known to drive TMA (systemic lupus erythematosus, antiphospholipid syndrome, scleroderma, and vasculitis).^6^^,^^7^ Finally, malignancy can drive both TMA and sarcoid-like reactions. However, no cancer has been detected on imaging, and tissue biopsies have been reassuring from this perspective. Sarcoid-like reactions can, in general, be difficult to distinguish from sarcoidosis, but without any known cancer or history of immunotherapy, it seems unlikely that this case is such a presentation.^8^

Given the widespread features of acute sarcoidosis and the sequence of events, a plausible explanation is that sarcoidosis represents the primary underlying pathology of this patient’s multiorgan dysfunction. We propose that sarcoidosis-driven hypercalcemia resulted in acute pancreatitis (reflected in the abdominal symptoms and raised amylase at presentation), which in turn led to TMA. Both sarcoidosis-induced hypercalcemia resulting in pancreatitis and this pathology leading to TMA are rare, but they have been described previously.^9^, ^10^, ^11^, ^12^, ^13^ Although two other cases of sarcoidosis and hemolytic uremic syndrome have been reported previously, our case is unique in the severity of the presentation and the lack of any renal granulomata, which would indicate co-presentation rather than a causal link.^14^^,^^15^

Collaborative input from a multidisciplinary team including hematologists, nephrologists, respiratory physicians, histopathologists, and thoracic radiologists was critical to managing this case. We would therefore advocate early specialist input in such unusual cases of multiorgan pathology to permit optimal treatment to be provided and good clinical outcomes achieved.
